# The Developmental Trajectory of the Operational Momentum Effect

**DOI:** 10.3389/fpsyg.2018.01062

**Published:** 2018-07-17

**Authors:** Pedro Pinheiro-Chagas, Daniele Didino, Vitor G. Haase, Guilherme Wood, André Knops

**Affiliations:** ^1^Cognitive Neuroimaging Unit, CEA DRF/I2BM, INSERM, Université Paris-Sud, Université Paris-Saclay, NeuroSpin Center, Orsay, France; ^2^Laboratory of Behavioral and Cognitive Neuroscience, Stanford Human Intracranial Cognitive Electrophysiology Program, Department of Neurology and Neurological Sciences, Stanford University, Stanford, CA, United States; ^3^Department of Psychology, Faculty of Life Sciences, Humboldt-Universität zu Berlin, Berlin, Germany; ^4^Developmental Neuropsychology Laboratory (LND), Department of Psychology, Universidade Federal de Minas Gerais, Belo Horizonte, Brazil; ^5^Programa de Pós-Graduação em Neurociências, Universidade Federal de Minas Gerais, Belo Horizonte, Brazil; ^6^Department of Psychology, Graduate Program in Psychology, Cognition and Behavior – Graduate Program in Neuroscience, Universidade Federal de Minas Gerais, Belo Horizonte, Brazil; ^7^Instituto Nacional de Ciência e Tecnologia sobre Comportamento, Cognição e Ensino, Universidade Federal de São Carlos, São Carlos, Brazil; ^8^Department of Psychology, University of Graz, Graz, Austria; ^9^BioTechMed-Graz, University of Graz, Graz, Austria; ^10^CNRS UMR 8240, Laboratory for the Psychology of Child Development and Education, Paris, France; ^11^University Paris Descartes, Sorbonne Paris Cité, Paris, France

**Keywords:** operational momentum, approximate addition, approximate subtraction, children, development, attentional shift account, compression account, heuristic account

## Abstract

Mental calculation is thought to be tightly related to visuospatial abilities. One of the strongest evidence for this link is the widely replicated operational momentum (OM) effect: the tendency to overestimate the result of additions and to underestimate the result of subtractions. Although the OM effect has been found in both infants and adults, no study has directly investigated its developmental trajectory until now. However, to fully understand the cognitive mechanisms lying at the core of the OM effect it is important to investigate its developmental dynamics. In the present study, we investigated the development of the OM effect in a group of 162 children from 8 to 12 years old. Participants had to select among five response alternatives the correct result of approximate addition and subtraction problems. Response alternatives were simultaneously presented on the screen at different locations. While no effect was observed for the youngest age group, children aged 9 and older showed a clear OM effect. Interestingly, the OM effect monotonically increased with age. The increase of the OM effect was accompanied by an increase in overall accuracy. That is, while younger children made more and non-systematic errors, older children made less but systematic errors. This monotonous increase of the OM effect with age is not predicted by the compression account (i.e., linear calculation performed on a compressed code). The attentional shift account, however, provides a possible explanation of these results based on the functional relationship between visuospatial attention and mental calculation and on the influence of formal schooling. We propose that the acquisition of arithmetical skills could reinforce the systematic reliance on the spatial mental number line and attentional mechanisms that control the displacement along this metric. Our results provide a step in the understanding of the mechanisms underlying approximate calculation and an important empirical constraint for current accounts on the origin of the OM effect.

## Introduction

Adults and children (Barth et al., 2006), and even infants (Wynn, 1992), are able to perform approximate mental calculation, which consists in the capacity to add or subtract numbers expressed in non-symbolic notations (e.g., dots). This skill requires to estimate the numerosity (i.e., cardinality) of two sets of elements and to encode it on an internal representation on which cognitive processes operate to generate the approximate outcome of the calculation. Growing evidence (McCrink et al., 2007; Pinhas and Fischer, 2008; Knops et al., 2009b; McCrink and Wynn, 2009; Lindemann and Tira, 2011; Chen and Verguts, 2012; Knops et al., 2013, 2014; Klein et al., 2014; Marghetis et al., 2014; Pinheiro-Chagas et al., 2017) shows that approximate addition and subtraction are subjected to an Operational Momentum (hereafter, OM) effect: results of addition are overestimated and results of subtraction are underestimated. Although an OM effect has been found in infants (McCrink and Wynn, 2009) and an inverse OM effect emerged in 6/7 years old children (Knops et al., 2013), no studies investigated the developmental trajectory of this effect. Therefore, it is still unclear how the OM effect evolves during the acquisition of formal mathematical knowledge. The relevance of the OM effect lies in the knowledge it provides regarding the cognitive mechanisms involved in the representation and the manipulation of non-symbolic numerical magnitudes. In this study, we aimed to measure how the OM effect evolves in children between 8 and 12 years of age. Moreover, the developmental trajectory of the OM effect can also provide evidence in favor of or against the current accounts proposed to explain this effect.

A prerequisite to perform approximate mental calculation is the capacity to estimate and manipulate numerical quantities, which is a phylogenetically ancient cognitive tool that humans share with other animals (Flombaum et al., 2005; Cantlon and Brannon, 2007; Piazza, 2010) and that arises early in life (Xu and Spelke, 2000; Izard et al., 2009). A widely accepted view (Dehaene, 1997) assumes that the mental representation of numerical magnitudes takes the form of an analog mental number line (hereafter, MNL). In the last decades, evidence has been collected to support the idea that on the MNL numerosities are spatially oriented in ascending order from left to right (Dehaene et al., 1993; Fias and Fischer, 2005; Hubbard et al., 2005; Rugani and Sartori, 2016; de Hevia et al., 2017). The SNARC effect (spatial numerical association of response codes; Dehaene et al., 1993) is often interpreted as evidence for the functional association between numbers and space: in a parity judgment tasks, where participant have to decide whether a displayed number is odd or even, left-hand responses are faster for relatively small number and right-hand responses for relatively large numbers (Dehaene et al., 1993; Fias and Fischer, 2005; Hubbard et al., 2005). Since the magnitude of the number is not relevant for the task, this spatial bias is assumed to reflect the automatic activation of the spatial mapping of magnitudes on the MNL (but for an alternative account see Santens and Gevers, 2008). The functional association between visuospatial processing and numerical magnitudes is additionally suggested by the mounting evidence showing that a shift of spatial attention can be induced by number processing (Sallilas et al., 2008; Ranzini et al., 2015, 2016; for a review see Fischer and Knops, 2014). It is worth noting that a functional association also emerges between shifts of spatial attention and mental arithmetic (Masson and Pesenti, 2014, 2016; Mathieu et al., 2016, 2017; Masson et al., 2017a,b). Moreover, converging evidence from behavioral (Izard and Dehaene, 2008), computational (Dehaene and Changeux, 1993), and neurophysiological studies (Nieder and Miller, 2003) suggests that the MNL is logarithmically compressed, which means that the representational overlap between adjacent quantities increases proportionally to their size, in accordance with the Weber–Fechner law (see Piazza et al., 2010).

Approximate calculation also follows the Weber–Fechner law (Barth et al., 2006; Dehaene, 2007), but it also shows an additional response bias, that is the OM effect. Three mutually not exclusive mechanisms have been proposed to explain the OM effect: attentional shift account, heuristic account, and compression account. However, none of them aimed to describe how this effect changes over development. Evidence shows that the neural network that supports mental calculation undergoes substantial functional changes during development and reaches an adult-like configuration only during adolescence (Rosenberg-Lee et al., 2011; Soltanlou et al., 2017, 2018; Arsalidou et al., 2018; Peters and De Smedt, 2018). Therefore, in order to fully understand the cognitive mechanisms lying at the core of the OM effect it is important to measure its developmental dynamics and to evaluate whether the current accounts are able to explain these age-related changes. In what follows, we introduce these accounts of the OM effect and discuss the developmental trajectories predicted by each of them.

It has been proposed that mental calculation is grounded in neural circuits that originally evolved for processing visuospatial information (Anderson, 2007; Dehaene and Cohen, 2007; Knops et al., 2009a). Moreover, various evidence supports the existence of a functional relationship between visuospatial attention (i.e., shift of spatial attention) and mental calculation (Masson and Pesenti, 2014, 2016; Mathieu et al., 2016, 2017; Masson et al., 2017a,b). In line with these studies, the *attentional shift account* proposes that the OM effect is the result of this functional relationship (McCrink et al., 2007; Knops et al., 2009b; Pinheiro-Chagas et al., 2017). The central assumption of the attentional shift account hypothesizes that non-symbolic addition and subtraction are implemented by shifting spatial attention on a spatially oriented MNL. During approximate calculation, the first operand is mapped on the MNL, then the attentional focus shifts from the current position (i.e., the point corresponding to the magnitude of the first operand) to a new position (i.e., the point corresponding to the magnitude of the result) by a distance corresponding to the magnitude of the second operand. The OM effect is produced by a bias in the attentional shift, that is the attentional focus moves too far along the MNL in the direction of the operation, generating an overestimation and an underestimation of the result of addition and subtraction, respectively. Strong evidence for the hypothesis that visuospatial attention is co-opted during mental calculation is provided by the overlap in the posterior superior parietal lobule (PSPL) of the neural activity associated with left/right saccades (i.e., visuospatial orientation) and mental calculation (Knops et al., 2009a).

McCrink and Wynn (2009) proposed the *heuristic account* to explain the finding that the OM effect also affects performance in 9 months old infants. This account assumes that infants adopted a simple heuristic to solve the problems: “if adding, accept larger outcomes,” “if subtracting, accept smaller outcomes.” For addition, this heuristic approach might encourage infants to perceive larger outcomes as more plausible compared smaller ones, and vice versa for subtraction. Recently, McCrink and Hubbard (2017) interpreted the finding that the OM effect increased in adults when available attentional resources were limited by dividing attention between two concurrent tasks as further evidence for the heuristics account. However, the heuristic account and the attentional shift account are deeply intertwined and can be considered as a single mechanism (i.e., heuristics-via-spatial-shifts account), that is the heuristic decision results from the visuospatial system (McCrink and Hubbard, 2017). Therefore, we will only focus on the attentional shift account, assuming that the two accounts provide equivalent predictions.

The attentional shift account has been developed to explain the OM effect in adults. Therefore, no predictions or hypotheses were proposed regarding how the attentional shifts on the MNL that accompany addition and subtraction emerge and whether they undergo substantial changes during development. Here, we propose that formal schooling (i.e., acquiring arithmetical skills) could reinforce (or even contribute to develop) the idea that addition is related with shifts toward larger numbers and subtraction toward smaller numbers. Namely, although mental calculation might be implemented as an attentional shift on the MNL before formal schooling, repeated exposition to spatial-numerical associations (e.g., the number line) might consolidate a systematic movement direction during the acquisition of arithmetical skills. Moreover, the systematic association between operations and results (i.e., when adding, the result is always larger than both operands; when subtracting, the result is always smaller than the first operand), that children are exposed to, could boost the attentional shift on the MNL. The influence of the attentional shift in the estimation of the result might increase with age and in turn a larger and more systematic bias would emerge. Therefore, one may predict an increasing OM effect during childhood. Moreover, it is worth noting that the co-opting of visuospatial attention during mental calculation seems to increase with age. In fact, significant functional changes associated with the neural activity elicited by symbolic arithmetic problem-solving have been found between 2nd and 3rd graders, that is 7–9 years old children (Rosenberg-Lee et al., 2011). During the processing of symbolic arithmetic problems, 3rd grade children showed greater activity in brain regions related to visuospatial attentional processes (posterior parietal cortex: intraparietal sulcus, superior parietal lobule, and angular gyrus) and high-order visual processing (ventral visual areas: lingual gyrus, right lateral occipital cortex, and right parahippocampal gyrus), compared to 2nd grade children.

The *compression account* has been proposed by McCrink et al. (2007) and deploys the logarithmic compression of the MNL to explain the OM effect. This compressed metric would generate a systematic operational bias in the direction of the operation due to the implementation of a linear arithmetic operation (i.e., addition or subtraction) on a logarithmically scaled mental representation. This mechanism acts in three steps. First, the operands are encoded as logarithmically compressed magnitudes on the MNL. Second, the logarithmic transformation is undone, which means that the operands are uncompressed to a linear scale. Third, the two uncompressed operands are added or subtracted. The OM effect results from the inaccuracy of the uncompression process. If the uncompression is ineffective the arithmetic operation is performed on logarithmic values and thus the generated outcome corresponds to an extreme overestimation or underestimation for addition and subtraction, respectively. If the uncompression is highly accurate the operation is performed on the linear scale, in which case the generated outcome corresponds (approximately) to the arithmetically correct result. A more plausible scenario is to assume that the actual degree of uncompression lies between these two extreme possibilities. An example can help describe this idea. If uncompression fails, adding two operands (e.g., 26 and 14) corresponds to adding their logarithmically compressed internal representation, that is log(26) ≈ 3.26 and log(14) ≈ 2.64, respectively. Since adding the logarithm of two numbers is equivalent to multiplying their linear values, the system generates an extreme overestimation of the correct result: log(26) + log(14) ≈ 5.9, which in linear scale corresponds to e^5.9^ ≈ 26 × 14 ≈ 364. However, the actual approximate addition performed by the system is much more accurate (see for example McCrink et al., 2007), and thus the uncompression is to some extent carried out and the generated outcome is much closer to the correct result. The same reasoning is valid to explain the mechanisms underpinning the underestimation of subtraction outcomes.

What developmental trajectory of the OM effect is expected according to the compression account? This account focuses on the logarithmic compression of the MNL. A large body of evidence suggests that the representational metric of the MNL shifts from a logarithmic to a linear scale during childhood (Siegler and Opfer, 2003; Siegler and Booth, 2004; Booth and Siegler, 2006, 2008; Laski and Siegler, 2007; Opfer and Siegler, 2007 but for a different interpretation see Barth and Paladino, 2011). The logarithmic-to-linear shift of the MNL implies that the compression of this magnitude representation decreases with age and probably with accumulation of experience in formal mathematics teaching. Therefore, the uncompression of the operands, performed before the approximate mental calculation, starts from a highly logarithmic scale in young children and from a more linear scale in adults. The degree of uncompression required to generate an accurate outcome is thus greater in young children and this in turn could lead to a stronger OM effect. The compression account therefore predicts that the size of the OM effect is higher in young children and decreases with age to reach an adult-like pattern in older children. It is worth noting that, as discussed below, the inverse OM effect (i.e., overestimation of subtraction problems) found in 6/7 years old children (Knops et al., 2013) already provides evidence against this account.

## Materials and Methods

The sample and the tasks analyzed in the present paper were administered to children as part of a larger study conducted in Brazil (for a more precise description of this larger study see Pinheiro-Chagas et al., 2014).

### Participants

One hundred seventy-two children from first to sixth grade were recruited from private and public schools in Brazil. Ten children were not able to perform non-symbolic numerical tasks, as shown by the fact that they failed to perform a non-symbolic number comparison task (this task is not reported here, for a more detailed description of this task see Pinheiro-Chagas et al., 2014). In that non-symbolic number comparison task, children had an accuracy less than 55% and a poor fit (*R*^2^< 0.2) in the estimation of the Weber fraction, and thus were excluded from the study. These ten children were also not included in the present analyses. The final sample consisted of 162 children (66 boys, 96 girls) between 8 and 12 years of age (mean = 9.7 years, *SD* = 1.1; 8 years old: 24 children, 9 years old: 54, 10 years old: 50, 11 years old: 20, 12 years old: 14). Informed written consent was obtained from the parents and oral consent from the children. This study was approved by the ethics review board of the Federal University of Minas Gerais, Brazil (COEP–UFMG).

All children performed above the 25th percentile in the spelling (mean = 110.08, *SD* = 8.13, range = [85, 126]) and arithmetic (mean = 108.92, *SD* = 11.41, range = [86, 134]) subtests of the TDE (Teste de Desempenho Escolar; Stein, 1994) and had a normal intelligence (mean = 110.61, *SD* = 10.55, range = [86, 134]), as measured by Raven’s Colored Progressive Matrices (Angelini et al., 1999).

### Tasks

#### Non-symbolic Estimation Task

In this task children were asked to estimate and report verbally the numerosity of a set of dots visually presented on a computer screen. Dots were displayed in black within a white circle, which was presented against a black background. The following numerosities were presented: 10, 16, 24, 32, 48, 56, or 64 dots. Each numerosity was presented five times (in a different configuration), resulting in a total of 35 trials. The same numerosity never appeared in consecutive trials. Each trial started with a fixation point (i.e., a white cross at the center of the screen) presented for 500 ms, followed by the onset of the set of dots which remained on the screen until spacebar was pressed or for up to 1000 ms. During the presentation of the dots, as soon as the child responded, the examiner, who was seated next to the child, pressed the spacebar on the keyboard and typed the child’s answer. The next trial started after an intertrial interval of 700 ms, which consisted of a black screen. Dots were displayed on the screen for up to 1000 ms only to prevent counting. To prevent the use of non-numerical features, total dot area was held constant across the trials and thus it could not be used as a clue to estimate the different numerosities. The average dot-size of the dots was selected so that the total area remained constant, but the dot-size of each dot could vary with a normal distribution with the mean selected to provide constant area across the trials. Therefore, while the average dot-size covaried negatively with numerosity, the dot-size of the single dots could not be used as a cue to evaluate the numerosity of the set. To avoid memorization effects due to the repetition of a specific numerosity, on each trial, the stimuli were randomly chosen from a set of 10 precomputed images with the given numerosity. To exclude extreme responses, the normalized mean estimated value was calculated for each child and each of the seven presented numerosities, then responses ±3 SD from the mean estimated value were considered outliers and excluded from the analysis (3.5% of the trials). Children’s number acuity was measured in term of individual mean coefficient of variation (i.e., separately for each numerosity, the ratio of standard deviation and mean chosen value).

#### Non-symbolic Approximate Calculation Task

This task has been adapted from Knops et al. (2013) study. Children were asked to solve approximate addition and subtraction problems with operands and proposed results presented in a non-symbolic notation (i.e., sets of dots). Problems are reported in **Table 1**. Eight addition and eight subtraction problems were generated. Both arithmetic operations had the same range of possible outcomes: 10, 16, 26, 40. To prevent the subjects from memorizing the problems, the operands were randomly “jittered” by adding a random value *r*, with r ∈ J and J = [-1, 0, 1]. For each correct outcome, seven response alternatives were generated as round (c × 2.5^i/3^), where *c* is the correct result and i = [-3,-2,-1,0,1,2,3]. To avoid a strategy of always selecting the response alternative falling in the middle of the proposed range, only five of the seven generated alternatives were presented in a trial (see **Table 1**). In one half of the trials, the presented responses were the upper five (henceforth, high range), and thus the correct outcome was the second smallest numerosity. In the other half, the presented responses were the lower five (henceforth, low range), and thus the correct outcome was the fourth smallest numerosity. Each trial was repeated twice and thus the total number of trials was 64: 2 operations (addition and subtraction) × 8 problems × 2 ranges (high and low) × 2 repetitions. To prevent the use of non-numerical features, total dot area and dot-size were manipulated as in the non-symbolic estimation task. To avoid memorization effects due to the repetition of a specific numerosity, on each trial, the stimuli were randomly chosen from a set of 10 precomputed images with the given numerosity. Trials without response and trials where the selected response was ±3 SD from the normalized mean chosen values (calculated combining addition and subtraction) were considered outliers and excluded from the analysis (3.1% of the trials). To analyze the OM effect, for each child and for each operation (addition vs. subtraction), mean chosen value, standard deviation, and coefficient of variation (i.e., the ratio of standard deviation and mean chosen value) were calculated for each of the four correct outcomes.

**Table 1:** Operands, correct outcome (C) and deviant (D) outcomes presented in the non-symbolic arithmetic problems.

Operands		Correct results and deviant proposed outcomes						
		1/2.5	1/1.8	1/1.4	1	1.4	1.8	2.5
**Addition**					
5	5	4	5	7	10	14	18	25
6	4	4	5	7	10	14	18	25
8	8	6	9	12	16	22	29	40
10	6	6	9	12	16	22	29	40
13	13	10	14	19	26	35	48	65
18	8	10	14	19	26	35	48	65
20	20	16	22	29	40	54	74	100
26	14	16	22	29	40	54	74	100
**Subtraction**					
16	6	4	5	7	10	14	18	25
20	10	4	5	7	10	14	18	25
24	8	6	9	12	16	22	29	40
32	16	6	9	12	16	22	29	40
40	14	10	14	19	26	35	48	65
52	26	10	14	19	26	35	48	65
62	22	16	22	29	40	54	74	100
80	40	16	22	29	40	54	74	100
**Range**					
Low		D	D	D	C	D		
High				D	C	D	D	D

*The last two rows report the set of outcomes presented in the two ranges.*

**FIGURE 1:**
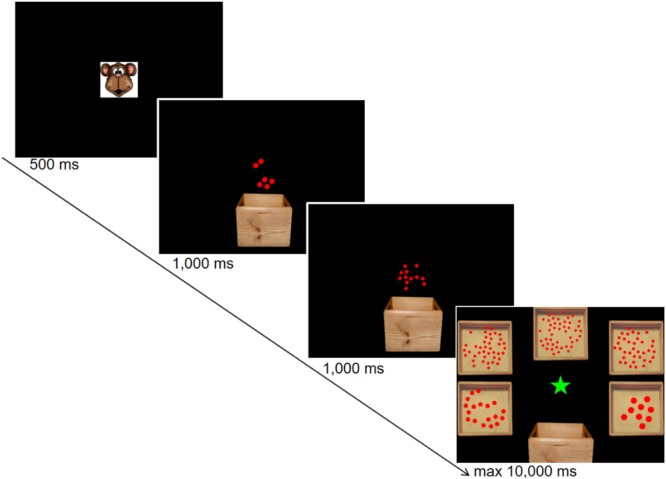
Trial sequence of the non-symbolic approximate calculation task. The example shows the screenshots from a non-symbolic addition trial. During the response period, the five response alternatives were presented in a circle-like shape around the center of the screen (i.e., green star) with two boxes on the left of the screen, two on the right, and one on the top.

To provide a child-friendly paradigm, problems were embedded in a story of a monkey having a box of balls (**Figure 1**). Each trial started with the drawing of the monkey’s face presented for 500 ms. After the offset of the monkey’s face, an empty brown box (against a black background) appeared at the bottom of the screen and a first set of red dots moved into the box. The first set of dots appeared at the top of the screen and moved toward the box until the dots disappeared inside it. For addition problems, a second set of red dots appeared at the top of the screen and disappeared inside the box in the same way. For subtraction problems, a set of red dots moved out of the box and disappeared at the top of the screen. Both for the first and the second sets, the duration of the dots movement (from the appearance to the disappearance) was 1000 ms. After the second set of dots disappeared, the box was replaced by the top-view of five boxes that contained five different sets of dots (i.e., five responses alternatives). Two boxes appeared on the left of the screen, two on the right, and one on the top. Children were asked to click with the left-key of the mouse on the box containing the set of dots which numerosity was the closest to the correct outcome of the operation. The beginning of the response active period was indicated by the appearance of the mouse pointer on top of a green star in the center of the screen. A training period consisting of two trials preceded the testing phase. In the training period, there was no time limit for the response and feedback was provided by a frame around the chosen box. The appearance of a green frame indicated a correct response, whereas a red frame indicated an incorrect response. If the response was incorrect, the child was asked to choose another box, and this procedure was repeated until the correct box was chosen. Before testing phase, the children were asked if they had understood the task, and if not, the training was repeated until they confirmed that they understood the task. In the testing phase, children had a maximum of 10,000 ms to select the box and the chosen box was indicated by a neutral blue frame (i.e., no feedback provided). Addition and subtraction problems were presented in different blocks counterbalanced across participants.

### Data Analysis

All analyses were performed using R-project software (R Core Team, 2015) and RStudio software (RStudio Team, 2015). In the following analyses, ANOVAs were Greenhouse-Geisser corrected (Greenhouse and Geisser, 1959) when the assumption of sphericity was violated; uncorrected degrees of freedom and epsilon values (𝜀GG) are reported. In the *post hoc* analyses all *p*-values have been corrected with Holm’s method (Holm, 1979). For the OM effect, effect sizes are reported following the recommendation of Lakens (2013). Additional analyses of children’s performance (absolute error) and of the operational bias (ratio) are reported in the Appendix A.

## Results

The results of all the ANOVAs performed on the tasks are reported in the Appendix B (Supplementary Table S2).

### Non-symbolic Estimation Task

**FIGURE 2:**
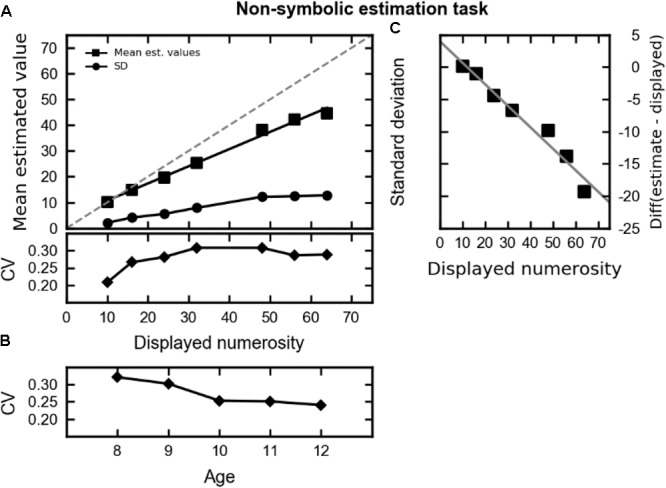
**(A)** The top part shows the mean chosen numerosities (squares; the black line represents the regression model) and standard deviation (circles) plotted against the displayed numerosity. The gray dashed line represents perfect performance. The lower part reports the mean CV (coefficients of variation) plotted against the displayed numerosity. **(B)** The mean CV plotted against the age groups. **(C)** The difference between chosen numerosity and displayed numerosity plotted against the displayed numerosity. The gray line represents a regression model between the variables.

The first analysis aims to evaluate the performance of children in the non-symbolic number estimation task. Mean chosen numerosity and CV were analyzed with a repeated measure ANOVA with displayed numerosity (i.e., 10, 16, 24, 32, 48, 56, and 64 dots) as within-subject factor and age (i.e., 8 to 12 years old) as between-subject factor. Mean chosen numerosities significantly increased with displayed numerosity [*F*(6,942) = 313.45, *p* < 0.001, 𝜀GG = 0.27, generalized η^2^ = 0.47]. However, as shown in **Figure 2**, and in line with adults’ behavior (Knops et al., 2014), children underestimated the larger displayed numerosities. To verify whether this pattern was statistically significant a repeated measure correlation (Bakdash and Marusich, 2017) was performed between numerical difference (chosen numerosity minus displayed numerosity) and displayed numerosity. There was a strong negative correlation between numerical difference and displayed numerosity [*r*_rm_(971) = -0.57, 95% CI = [-0.61, -0.53], *p* < 0.001], that is the discrepancy between displayed and chosen values increased with numerosity (**Figure 2**). In the ANOVA, neither the main effect of age nor the interaction was significant.

On the basis of the assumption that mental numerosity representation is subjected to the Weber–Fechner law, the CV should not covary with displayed numerosity (i.e., the CV should be constant across numerosities). As shown in **Figure 2**, the CV is lowest for the displayed numerosity 10 and increases with displayed numerosity [*F*(6,942) = 11.04, *p* < 0.001, 𝜀GG = 0.92, $\eta_{G}^{2}$ = 0.05]. To further explore the relationship between CV and displayed numerosity, we performed a repeated measure correlation (Bakdash and Marusich, 2017) between these two variables. A weak positive correlation emerged [*r*_rm_(971) = 0.16, 95% CI = [0.10, 0.22], *p* < 0.001], showing that the CV slightly increases with displayed numerosity. The ANOVA also revealed that the CV decreased with age [*F*(4,157) = 5.26, *p* < 0.001, $\eta_{G}^{2}$ = 0.04; see **Figure 2**] but no interaction was observed [*F*(24, 942) < 1]. This indicates that the overall accuracy increased with age.

To account for putative effects of inflated variance due to small number of trials in each displayed numerosity, we repeated these analyses using the *z*-transformed scores. For both mean chosen numerosity and CV, we calculated the standardized z-scores over all displayed numerosity for each child. The mean *z*-scores were entered into a repeated measure ANOVA with age as between-subject factor. Similar results emerged. In fact, age significantly influenced CV [*F*(4,157) = 5.37, *p* < 0.001] but not mean chosen numerosity [*F*(4,157) < 1].

### Distribution of Responses in Approximate Addition and Subtraction

**FIGURE 3:**
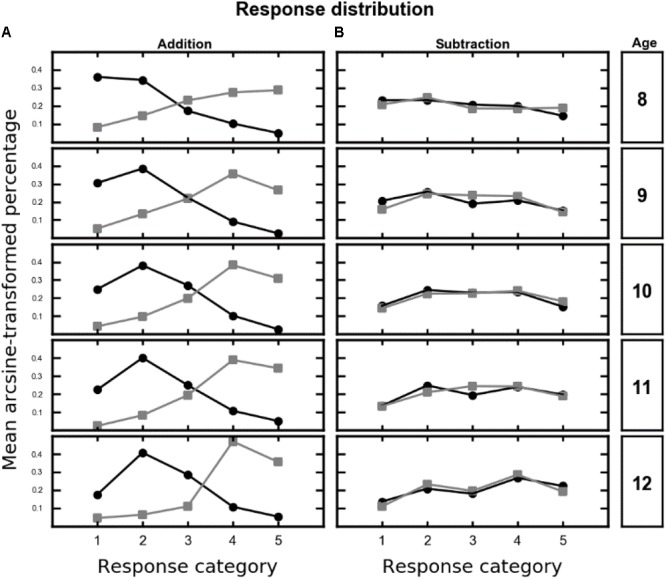
Mean (arcsine-transformed) percentage of choice across the response category (*x*-axis) as a function of range (high: black circles, low: gray squares) and age (from 8 to 12, rows), for addition **(A)** and subtraction **(B)**. For high range the correct outcome is the response category 2, for low range the correct outcome is the response category 4.

In each trial, the set of five proposed alternatives was sampled from either the lower range of responses (alternatives from 1 to 5, see **Table 1**) or the higher range (alternatives from 3 to 7, see **Table 1**). Therefore, the correct outcome was either the second (high range) and the fourth (low range) smaller proposed alternative. If children were able to solve the calculation, the response pattern should show a non-flat distribution centered on the correct outcome (i.e., second or fourth smaller alternative for high and low range, respectively). Mean (arcsine-transformed) percentage of choice was analyzed with a repeated-measure ANOVA with response category (i.e., 1 to 5), range (i.e., low vs. high), and operation (i.e., addition vs. subtraction) as within-subject factors and age (i.e., 8 to 12 years old) as between-subject factor. Results are reported in Supplementary Table S2 (see Appendix B). In particular, both the operation × range × response category interaction [*F*(4,628) = 141.89, *p* < 0.001, 𝜀GG = 0.95, generalized η^2^ = 0.16] and the age × range × response category interaction [*F*(16,628) = 1.71, *p* = 0.048, 𝜀GG = 0.89, generalized η^2^ = 0.01] were significant. Moreover, the four-way interaction showed a tendency toward significance [*F*(16,628) = 1.54, *p* = 0.085, 𝜀GG = 0.95, generalized η^2^ < 0.01]. The tendency of the four-way interaction and **Figure 3** suggest that the performance was different in the two operations. Therefore, to further explore this pattern, two additional ANOVAs were performed on mean percentage of choice with response category and range as within-subject factors and age as between-subject factor, separately for addition and subtraction.

For addition, the main effect of response category was significant [*F*(4,628) = 22.06, *p* < 0.001, 𝜀GG = 0.89, generalized η^2^ = 0.06]. Moreover, the age × response category [*F*(16,628) = 2.19, *p* = 0.007, 𝜀GG = 0.89, generalized η^2^ = 0.03], the range × response category interaction [*F*(4,628) = 223.06, *p* < 0.001, 𝜀GG = 0.87, generalized η^2^ = 0.43] and the three-way interaction [*F*(16,628) = 2.07, *p* = 0.012, 𝜀GG = 0.87, generalized η^2^ = 0.03] were significant (**Figure 3**).

For subtraction, only the main effect of response category [*F*(4,628) = 19.18, *p* < 0.001, 𝜀GG = 0.89, generalized η^2^ = 0.07] and the age × response category interaction [*F*(16,628) = 2.02, *p* = 0.014, 𝜀GG = 0.89, generalized η^2^ = 0.03] were significant, whereas neither the range × response category interaction [*F*(4,628) = 2.07, *p* = 0.087] nor the three-way interaction [*F*(16,628) < 1] reached significance (**Figure 3**). The response distribution for subtraction was flatter, showing that children found more difficult to perform approximate subtraction.

### Children’s Performance in Approximate Calculation

**FIGURE 4:**
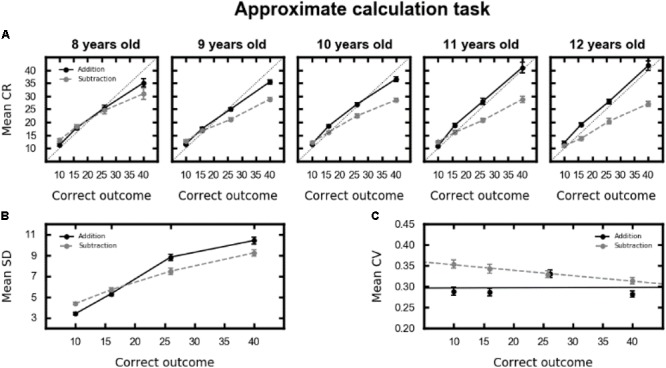
**(A)** Mean chosen response (CR) as a function of correct outcome (*x*-axis), operation (addition in black, subtraction in gray), and age (columns). The black dotted lines represent perfect performance. **(B)** Mean standard deviation (SD) as a function of correct outcome (*x*-axis) and operation (addition in black, subtraction in gray), collapsed across all ages. **(C)** Mean coefficients of variation (CV) as a function of correct outcome (*x*-axis) and operation (addition in black, subtraction in gray, the lines represent the regression models), collapsed across all ages. In all plots, error bars represent the standard error of the mean.

In order to evaluate children’s performance in approximate addition and subtraction, mean chosen response and standard deviation were analyzed with a repeated-measure ANOVA with correct outcome (i.e., 10, 16, 26, and 40) and operation (i.e., addition vs. subtraction) as within-subject factors and age (i.e., 8–12 years old) as between-subject factor. For mean chosen response, the main effect of correct outcome was significant [*F*(3,471) = 1685.80, *p* < 0.001, 𝜀GG = 0.60, $\eta_{G}^{2}$ = 0.76]. Mean chosen responses increased with correct outcome (mean responses: 12.0, 17.3, 24.1, and 32.9 for the outcomes 10, 16, 26, and 40, respectively). Mean chosen responses were greater for addition (mean = 23.2) than for subtraction (mean = 19.9) [*F*(1,157) = 93.49, *p* < 0.001, $\eta_{G}^{2}$ = 0.12]. Moreover, all the two-way interactions were significant: correct outcome × operation [*F*(3,471) = 131.81, *p* < 0.001, 𝜀GG = 0.72, $\eta_{G}^{2}$ = 0.12], correct outcome × age [*F*(12,471) = 2.03, *p* = 0.049, 𝜀GG = 0.60, $\eta_{G}^{2}$ = 0.01], operation × age [*F*(4,157) = 6.24, *p* < 0.001, $\eta_{G}^{2}$ = 0.04]. Interestingly, the three-way interaction was also significant [*F*(12,471) = 2.78, *p* = 0.004, 𝜀GG = 0.72, $\eta_{G}^{2}$ = 0.01]. As shown in **Figure 4**, mean chosen values were overestimated for addition compared to subtraction, and this difference was greater for larger numerosities and increased with age. This pattern reflects the OM effect and will be further investigated in the following section.

Standard deviation significantly increased with correct outcome [*F*(3,471) = 275.66, *p* < 0.001, 𝜀GG = 0.82, $\eta_{G}^{2}$ = 0.35]. However, this increase followed a different pattern in the two operations, as shown by the correct outcome by operation interaction [*F*(3,471) = 18.17, *p* < 0.001, 𝜀GG = 0.88, $\eta_{G}^{2}$ = 0.02], see **Figure 4**. No other main effects or interactions were significant.

To investigate whether children’s mental numerosity representation follows Weber–Fechner law, a third ANOVA was performed on CV with correct outcome and operation as within-subject factors and age as between-subject factor. The main effect of correct outcome was significant [*F*(3,471) = 5.88, *p* < 0.001, 𝜀GG = 0.90, $\eta_{G}^{2}$ = 0.01] [outcomes 10: mean CV (*SD*) = 0.32 (0.09); outcome 16: 0.31 (0.09); outcome 26: 0.33 (0.09); outcome 40: 0.30 (0.07)]. Moreover, the CV was also significantly smaller for addition (mean = 0.30, *SD* = 0.08) than for subtraction (mean = 0.33, *SD* = 0.08) [*F*(1,157) = 30.28, *p* < 0.001, $\eta_{G}^{2}$ = 0.03]. Finally, the interaction between correct outcome and operation was significant [*F*(3,471) = 7.46, *p* < 0.001, 𝜀GG = 0.96, $\eta_{G}^{2}$ = 0.01], see **Figure 4**. To further investigate this interaction, we performed a repeated measure correlation between correct outcome and CV, separately for each operation. For addition, no correlation emerged between CV and correct outcome [*r*_rm_(485) = 0.005, 95% CI = [-0.08, 0.09], *p* = 0.91]. For subtraction, a weak negative correlation emerged [*r*_rm_(485) = -0.17, 95% CI = [-0.25, -0.08], *p* < 0.001], showing that mean CV slightly decreased with correct outcome, and thus the variability of the chosen response did not increase proportionally with the mean of the chosen response. These results are not perfectly consistent with the assumption that the underlying mental numerosity representation follows the Weber–Fechner law. However, since the CV did not covary with correct outcome in addition and only weakly correlated with it in subtraction (explained variance: 2.89%), the overall performance did not substantially deviate from this assumption.

### Operational Momentum Effect

**FIGURE 5:**
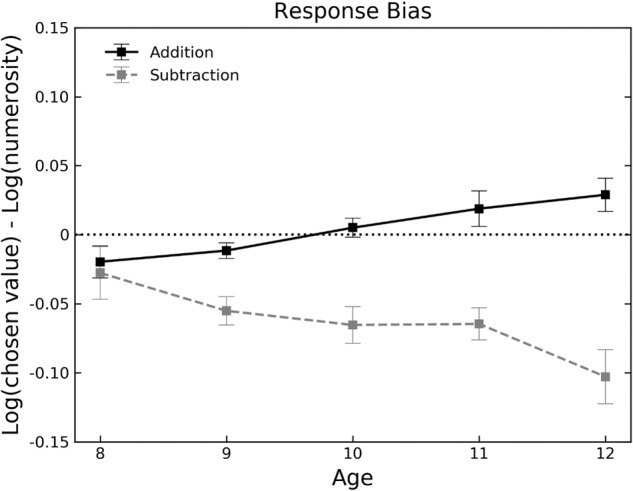
Mean response bias (i.e., difference between the logarithm of the chosen response and the logarithm of the correct outcome) as a function of age and operation (addition in black, subtraction in gray dashed). Error bars represent the standard error of the mean. The horizontal dotted line represents no bias.

To investigate the developmental trajectory of the OM effect, the mean response bias was analyzed with a repeated-measure ANOVA with operation as within-subject factor and age as between-subject factor. Response bias was calculated as the mean difference between the logarithm of the chosen response and the logarithm of the correct outcome. Response bias was significantly different between addition (-0.0004, *SD* = 0.05) and subtraction (-0.06, *SD* = 0.08) [*F*(1,157) = 60.2, *p* < 0.001, $\eta_{G}^{2}$ = 0.17]. The age by operation interaction was also significant [*F*(4,157) = 4.45, *p* = 0.002, $\eta_{G}^{2}$ = 0.06]. As shown in **Figure 5**, the OM effect monotonically increased with age^1^, from no effect for younger children to a strong effect for older children (see **Table 2** for *post hoc* comparison and effect sizes). To further explore the addition and subtraction response biases separately, a second set of one-sample *t*-tests have been performed to evaluate whether they significantly differed from zero (biases significantly different from zero are shown in bold in **Table 2**). As shown in the table, only subtraction biases for the age groups from 9 to 12 were significantly different from zero [all *t*s < -4.97, all *p*s < 0.01].

**Table 2:** *T*-tests comparing the response bias between addition and subtraction in the different age groups.

Age group	*N*	Addition		Subtraction		*t*	df	*p*-value	Cohen’s *d*_z_	Hedges’ *g*_av_
		Mean	*SD*	Mean	*SD*					
8	24	–0.020	0.057	–0.028	0.094	0.4	23	>0.1	0.08	0.10
9	54	–0.012	0.041	–**0.055**	0.075	3.61	53	0.005	0.49	0.71
10	50	0.005	0.048	–**0.065**	0.093	4.55	49	<0.001	0.64	0.94
11	20	0.019	0.058	–**0.065**	0.052	4.52	19	0.002	1.01	1.46
12	14	0.029	0.045	–**0.103**	0.073	5.04	13	0.002	1.35	2.04

*All p-values have been corrected with Holm’s method. For the calculation of the effect sizes (Cohen’s d_*z*_ and Hedges’ g_*av*_) refers to Lakens (2013). Mean response biases significantly different from zero (i.e., one-sample t-tests, separately computed for each operation and age group) are in bold, all ps < 0.01.*

In Appendix A, we report an additional set of analyses that by and large confirms these findings.

## Discussion

This study aimed to investigate the developmental trajectory of the OM effect in children aged from 8 to 12 years old and to assess whether the current accounts are able to predict these age-related changes. Concerning the non-symbolic estimation task, consistent with previous research (Izard and Dehaene, 2008; Knops et al., 2014; but for overestimation see Mejias and Schiltz, 2013), children underestimated the cardinality of displayed numerosities and this underestimation increased with numerosity. Although the CV significantly increased with numerosity, the correlation between the two variable was weak (*r*_rm_ = 0.16). Moreover, both mean estimated values and standard deviation increased with displayed numerosity. This suggests that children’s performance was by and large well captured by Weber–Fechner law, even if the CV was not perfectly linear across the entire numerical range. In line with previous findings that suggest that the Weber fraction decreases with age (Piazza et al., 2010; Halberda et al., 2012), the coefficient of variation also significantly decreased with age. Deviations may be due to non-numerical features of the stimulus set, for example. Further studies are needed to fully explain these inconsistencies.

In the approximate addition task, the distribution of responses clearly peaked around the correct outcome showing that children were able to solve these problems. The response distribution for subtraction problems, however, showed a different pattern. The distribution was flat for younger children (8 years old, see **Figure 3**) and in general the two ranges (low vs. high, see **Table 1**) were almost overlapped. Therefore, children found subtraction problems more difficult to solve compared to addition problems, in line with adults (Knops et al., 2009b). However, for subtraction problems, the significant main effect of response category and **Figure 3** suggest that children (at least in the age groups from 9 to 12) did not respond at random but rather selected more often values in the center of the response category range (i.e., 2, 3, 4) compared to the extremes (i.e., 1 and 5). This suggests that children might have used a different strategy to perform subtraction compared to addition. Despite the lower performance on subtractions problems, a clear OM effect emerged in our sample. Importantly, for addition the increase of the OM effect was accompanied by an increase in overall accuracy (see **Figure 3**). That is, while younger children made more and non-systematic errors, older children made less but systematic errors. Interestingly, the OM effect monotonically increases with age. While no effect was present in younger children (8 years-olds), the OM effect (i.e., the relative difference between the estimated responses in addition and subtraction) increased with age. In what follows, we first summarize the findings related to the evolution of the OM effect during childhood, and then we will discuss the implications of these findings for the current accounts of the OM effect (i.e., compression account and attentional shift account).

McCrink and Wynn (2009) found that 9 months old infants exhibit an OM effect similar to that found in adults. Although the similarity between the OM effect found in infants (McCrink and Wynn, 2009) and adults (McCrink et al., 2007; Knops et al., 2009b) would suggest that the OM effect results from inherited mechanisms (since infants are not yet affected by cultural practices) and remains constant during development, a more complex pattern emerges if we consider a previous study (Knops et al., 2013) and the findings reported in the current paper. In fact, contrary to the expected continuity of the OM effect during development, Knops et al. (2013) found an inverse OM effect in 6/7 years old children: subtraction was significantly overestimated compared to addition. Finally, our results showed a monotonic increase of the OM effect with age. This complex developmental pattern indicates that the evolution of the OM effect is not linear. In fact, a standard OM effect emerges in infants (McCrink and Wynn, 2009), an inverse OM effect was found in 6/7 years old children (Knops et al., 2013), and our results show no OM in 8 years old children and a monotonically increasing OM effect from 9 to 12 years old.

How well do the current accounts predict the developmental-related changes of the OM effect? The *compression account* (McCrink et al., 2007) predicts that, due the logarithmic-to-linear shift of the MNL during childhood (Siegler and Opfer, 2003; Siegler and Booth, 2004; Booth and Siegler, 2006, 2008; Laski and Siegler, 2007; Opfer and Siegler, 2007; but for a different perspective see Barth and Paladino, 2011), the OM effect decreases with age. Our result clearly points in the opposite direction showing an increase of the OM effect.

In line with the recycling theory (Dehaene and Cohen, 2007; see also the redeployment theory, Anderson, 2007), which proposes that arithmetic calculation is grounded on the recycling of neural circuits that originally evolved for processing visuospatial information, the *attentional shift account* assumes that the OM effect is driven by the functional relationship between visuospatial attention and mental arithmetic. Strong evidence for the idea that visuospatial attention is co-opted during mental calculation is provided by the fact that the neural activity associated with left/right saccades (i.e., visuospatial orientation) and mental calculation overlap in the posterior superior parietal lobule (Knops et al., 2009a). Using fMRI data, these authors showed that a multivariate classifier algorithm trained to classify the neural activity elicited by leftward and rightward saccades was able to generalize to approximate arithmetic. Without further training, this algorithm was able to distinguish between addition and subtraction by classifying approximate additions as rightward saccades. The activation of the same neural areas during rightward saccades and approximate addition speaks in favor of the recruitment of attentional shift mechanisms during mental calculation. This hypothesis stipulates a functional coupling between eye movements and arithmetic. A recent study provided confirmatory evidence for this notion (Klein et al., 2014). Participants’ eye movements after the first saccade were observed to move to the right during addition problems and to the left in subtraction problems when asked to indicate the location of the result on a labeled line (Klein et al., 2014). Moreover, the redeployment of visuospatial attention during mental calculation seems to be enhanced during formal schooling (Rosenberg-Lee et al., 2011). Finally, on the behavioral level, too, even if spatial-numerical association already emerges in preschoolers, the evidence is mixed. For example, White et al. (2012) found that the SNARC effect emerged during the 2nd year of schooling in British students, that is at around 7 years of age, while 6-year-olds did not show a significant SNARC effect (see also Gibson and Maurer, 2016). Moreover, Yang et al. (2014) found a SNARC effect in kindergarteners (age range: 4.8–6.4 years), 2nd, 3rd, 5th, and 6th graders, while 1st and 4th graders did not show a significant effect (see also Patro and Haman, 2012). Hoffmann et al. (2013) also found mixed evidence for the emergence of the SNARC effect. While all children in the second-term (mean age: 5.8 years old) showed a SNARC effect, in the first-term group (5.5 years old) the effect emerged when a magnitude comparison task preceded a digit color judgment task but not when the task order was inverted. Moreover, in the magnitude comparison task the size of the SNARC effect was related to proficiency with Arabic numbers. This developmental pattern suggests that the spatial-numerical association is still immature in young children. We propose that formal schooling could bolster spatial-numerical associations and hence reinforce movement direction during addition (toward larger numbers) and subtraction (toward smaller numbers). Attentional shifts may implement the core cognitive function to carry out the shifts along the spatial mental number representation and may be affected in at least two ways by the emerging spatial-numerical associations. Either the amount of displacement in the direction of the operation on the MNL increases (i.e., generate a larger and/or more systematic bias) or the variance of displacement is reduced while the overall amplitude remains constant. Therefore, the attentional shift account predicts an increasing OM effect during childhood. Consistent with this prediction, we found a monotonous increase of the OM effect with age.

Although the attentional shift account is consistent with our results, a more complex picture emerges if the results from previous studies are taken into account. In fact, the inverse OM effect found in 6/7 years old children (Knops et al., 2013) is neither explained nor predicted by this account. However, Knops et al. (2013) showed that the direction of the OM effect was related to reorienting attention in a Posner paradigm. The reorientation effect was calculated as the difference in reaction times between valid (i.e., the target stimulus appeared on the left or right of a bidirectional arrow previously presented in the center of the screen) and invalid trials (i.e., the target stimulus appeared opposite the pointing direction of a single-headed arrow). In their study, children who exhibited a smaller reorientation effect (i.e., more proficient to reorient attention after an invalid cue) also had a more regular OM effect (i.e., addition overestimated compare to subtraction). As those authors suggested, it can be hypothesized that the OM effect relies on a fully developed attentional system and on a robust functional association between visuospatial attention and mental calculation. Alternatively, it may suggest that inhibitory control of saccadic eye movements plays a crucial role for the association between attention and arithmetic. We can only speculate as to why an inverse OM effect emerges in 6/7 years old children and the youngest age group of our sample does not show any effect. The more immature attentional system (Rueda et al., 2004; Konrad et al., 2005) and the weaker functional connection between visuospatial processing and mental calculation (Rosenberg-Lee et al., 2011) might be at the origin of the inverse OM effect and its absence in younger children. Namely, the implementation of approximate addition and subtraction would not be yet supported by operation-specific, systematic attentional shifts on the MNL that produce misestimation in the direction of the operation.

The presence of a standard OM effect in infants (McCrink and Wynn, 2009) challenges the idea that the OM effect monotonically increases during childhood due to the consolidation of the engagement of visuospatial processing during mental calculation. However, this contradiction strongly relies on the idea that the development of cognitive performance always reflects linear developmental trajectories. However, as put forward by Siegler (1996), behavior may reflect the prevalence of heuristics and biases that wax and wane over time. That is, while infants may respond according to a given heuristic, the very same heuristic may be less influential during later periods in life. In children, performance in approximate calculation tasks may be performed with the support of the visuospatial system (i.e., the shift of the attentional focus on the MNL), while in infants the heuristic decision may result from simpler processes rather than from more sophisticated attentional mechanisms. Namely, in children (or adults) and infants the heuristic decision might result from different mechanisms. However, more evidence on the development of the OM effect is needed to unravel the cognitive mechanisms that drive the OM at different ages.

This study has some limitations. First, children’s performance in subtraction was low compared to addition. The higher difficulty to estimate the result of approximate subtraction could be due to the use of different strategies to perform the two operations. To better understand how children perform approximate calculation, future research should further investigate this difference in performance. Second, despite the fairly large sample, 6/7 years old children were not included, that is the age group that showed the inverse OM effect. Future studies should include a larger age range in order to confirm the inverse OM effect and to further investigate the development of this effect. Third, we did not include any task to measure visuospatial attention. Future studies should investigate whether there is a correlation between the developmental trajectories of visuospatial attention and of the OM effect. Finally, the effect of education is also accompanied by the maturation of neural network that supports mental calculation. In the analysis we focused on age, future research, however, should also disentangle the influence of age (neural maturation) and grade (education) on the OM effect. These two independent factors could make distinct contribution at various stages of development.

To sum up, we provided a novel finding on the developmental trajectory of the OM effect in children from 8 to 12 years old. The OM effect monotonically increases with age. This developmental pattern is inconsistent with the compression account. On the other hand, the attentional shift account provides a possible explanation of these results based on the functional relationship between visuospatial attention and mental calculation and on the effect of the acquisition of arithmetical skills during formal schooling. The attentional shift account leads to new predictions about a correlation between visuospatial processing and mental calculation which can be addressed in future studies. Our results provide an important empirical constraint to further explore the origin of the OM effect.

## Ethics Statement

This study was carried out in accordance with the recommendations of ethics review board of the Federal University of Minas Gerais, Brazil (COEP–UFMG) with written informed consent from all subjects. All subjects gave written informed consent in accordance with the Declaration of Helsinki. Informed written consent was obtained from the parents and oral consent from the children. The protocol was approved by the ethics review board of the Federal University of Minas Gerais, Brazil (COEP–UFMG).

## Author Contributions

PP-C, VH, GW, and AK designed the research. PP-C performed the research. DD and PP-C analyzed the data. DD drafted the manuscript. DD, PP-C, AK, VH, and GW contributed to write and revise the paper.

## Conflict of Interest Statement

The authors declare that the research was conducted in the absence of any commercial or financial relationships that could be construed as a potential conflict of interest.

## Footnotes

1 Since the sample size is unequal in the different age groups, we also performed two Spearman’s correlation analyses between mean response bias and age (in months), separately for addition and subtraction. For addition, there was significant positive correlation [*r* = 0.31, *p* < 0.001]. For subtraction, there was significant negative correlation [*r* = -0.24, *p* = 0.002].
