# Supplementary material for: The Developmental Trajectory of the Operational Momentum Effect
Source: Front Psychol. 2018 Jul 17;9:1062. doi: 10.3389/fpsyg.2018.01062 (PMC6056750; doi:10.3389/fpsyg.2018.01062)
Supplement: Supplementary file 1 [file Data_Sheet_1.docx]

**APPENDIX A – Additional analysis**

The following analyses aimed to further explore the performance of children in the non-symbolic approximate calculation task and the direction of the operational bias (for a similar analysis see Mioni et al., 2014; see also Brown, 1985, 1997; Glicksohn & Hadad, 2012). Performance was measured with the absolute error (AE), calculated as the absolute value of the difference between the mean chosen response (CR) and the correct outcome (CO), divided by the correct outcome: AE = |CR – CO| / CO (perfect performance: AE = 0; the greater the AE, the lower the performance). The direction of the operational bias was measured with ratio (R), calculated by dividing the mean chosen response by the correct outcome: R = CR/CO (perfect performance: R = 1; overestimation: R > 1; underestimation: R < 1). In the post-hoc analyses all *p*-values have been corrected with Holm’s method (Holm, 1979). Both absolute error and ratio were analysed with a repeated-measure ANOVA with operation (i.e., addition vs. subtraction) as within-subject factors and age (i.e., 8 to 12 years old) as between-subject factor.

**Absolute error**. Only the main effect of operation was significant [*F*(1,157) = 97.10, *p* < 0.001, generalised eta^2^ = 0.22]. The AE was significantly lower for addition (AE = 0.17, SD = 0.07) compared to subtraction (AE = 0.24, SD = 0.08). This result confirms that children found subtraction problems more difficult to solve compared to addition problems.

**Ratio**. The main effect of operation was significant [*F*(1,157) = 34.77, *p* < 0.001, generalised eta^2^ = 0.10]. This analysis confirms that addition (R = 1.06, SD = 0.13) was overestimated compared to subtraction (R = 0.96, SD = 0.17). The operation X age interaction was also significant [*F*(4,157) = 5.26, *p* < 0.001, generalised eta^2^ = 0.06], reflecting the fact that the OM effect increased with age (**Figure S1** and **Table S1**).


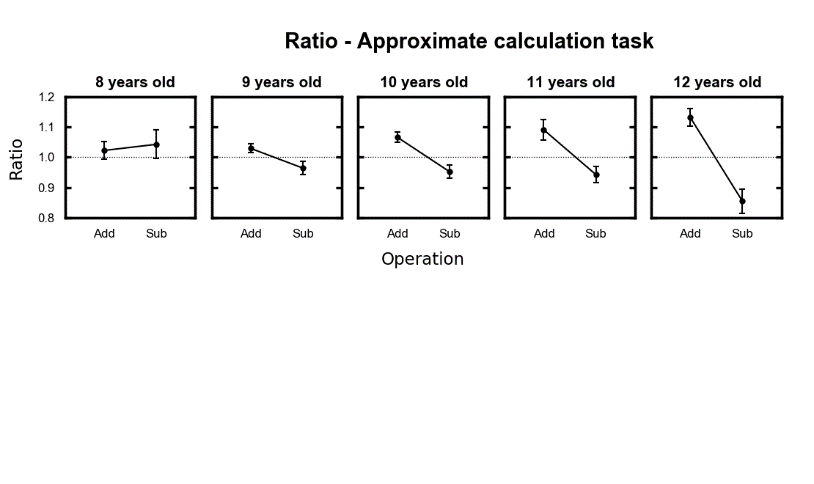


**FIGURE S1**. Mean ratio as a function of operation (x axis) and age (columns) The horizontal grey dotted lines represent perfect performance. In all plots, error bars represent the standard error of the mean.

**Table S1.** T-tests comparing the mean ratios between addition and subtraction in the different age groups.

| Age group | N | Addition | | Subtraction | | *t* | df | *p*-value | Cohen’s d_z_ | Hedges g_av_ |
| --- | --- | --- | --- | --- | --- | --- | --- | --- | --- | --- |
|  |  | Mean | SD | Mean | SD |  |  |  |  |  |
| 8 | 24 | 1.023 | 0.142 | 1.043 | 0.230 | −0.44 | 23 | > 0.1 | 0.09 | 0.10 |
| 9 | 54 | 1.029 | 0.105 | 0.964 | 0.163 | 2.37 | 53 | > 0.1 | 0.32 | 0.47 |
| 10 | 50 | **1.066 ^**^** | 0.121 | 0.953 | 0.158 | 3.97 | 49 | 0.004 | 0.56 | 0.80 |
| 11 | 20 | 1.091 **^+^** | 0.151 | 0.943 | 0.121 | 3.16 | 19 | 0.052 | 0.71 | 1.04 |
| 12 | 14 | **1.132 ^**^** | 0.107 | **0.855 ^*^** | 0.151 | 4.93 | 13 | 0.004 | 1.32 | 1.99 |

All *p*-values have been corrected with Holm method. For the calculation of the effect sizes (Cohen’s d_z_ and Hedges’ g_av_) refers to Lakens (2013). Mean ratios significantly different from one (i.e., one-sample t-tests, separately computed for each operation and age group) are in bold (^+^ *p* < 0.1, * *p* < 0.05, ** *p* < 0.01).

**APPENDIX B – Results of all ANOVAs performed**

**Table S2.** Results of the ANOVAs performed on the non-symbolic estimation task and the non-symbolic approximate calculation task. ANOVAs were Greenhouse-Geisser corrected (Greenhouse & Geisser, 1959) when the assumption of sphericity was violated; corrected *p*-value and uncorrected degrees of freedom and epsilon values (εGG) are reported. Effect sizes are expressed as generalized eta^2^.

| Dependent variable & Factors | *F* | df | *p*-value | εGG | generalised  eta^2^ |
| --- | --- | --- | --- | --- | --- |
|  |  |  |  |  |  |
| **Mean chosen numerosity (estimation task)** | | | | | |
| Displayed numerosity | 313.45 | 6, 942 | < .001 | .27 | .47 |
| Age | < 1 | 4, 157 | > .1 | - | < .01 |
| Displayed numerosity X Age | < 1 | 24, 942 | > .1 | .27 | < .01 |
| **CV (estimation task)** | | | | | |
| Displayed numerosity | 11.04 | 6, 942 | < .001 | .92 | .05 |
| Age | 5.26 | 4, 157 | < .001 | - | .04 |
| Displayed numerosity X Age | < 1 | 24, 942 | > .1 | .92 | .02 |
| **Mean (arcsine-transformed) percentage of choice (approximate calculation)** | | | | | |
| Response category | 20.84 | 4, 628 | < .001 | .90 | .06 |
| Range | < 1 | 1, 157 | > .1 | - | < .01 |
| Operation | 180.57 | 1, 157 | < .001 | - | < .01 |
| Age | 1.60 | 4, 157 | > .1 | - | < .01 |
| Response category X Range | 148.71 | 4, 628 | < .001 | .89 | .19 |
| Response category X Operation | 8.82 | 4, 628 | < .001 | .95 | < .01 |
| Response category X Age | 2.13 | 16, 628 | .009 | .89 | .03 |
| Range X Operation | 1.16 | 1, 157 | > .1 | - | < .01 |
| Range X Age | 1.39 | 4, 157 | > .1 | - | < .01 |
| Operation X Age | 2.12 | 4, 157 | .081 | - | < .01 |
| Response category X Range X Operation | 141.89 | 4, 628 | < .001 | .95 | .16 |
| Response category X Range X Age | 1.71 | 16, 628 | .048 | .89 | .01 |
| Response category X Operation X Age | 1.07 | 16, 628 | > .1 | .95 | < .01 |
| Range X Operation X Age | 2.00 | 4, 157 | .097 | - | < .01 |
| Response category X Range X Operation X Age | 1.54 | 16, 628 | .085 | .95 | < .01 |
| **Mean (arcsine-transformed) percentage of choice (approximate addition)** | | | | | |
| Response category | 22.06 | 4, 628 | < .001 | .89 | .06 |
| Range | 1.11 | 1, 157 | > .1 | - | < .01 |
| Age | 1.90 | 4, 157 | > .1 | - | < .01 |
| Response category X Range | 223.06 | 4, 628 | < .001 | .87 | .43 |
| Response category X Age | 2.19 | 16, 628 | .007 | .89 | .03 |
| Range X Age | 1.69 | 4, 157 | > .1 | - | < .01 |
| Response category X Range X Age | 2.07 | 16, 628 | .012 | .87 | .03 |
| **Mean (arcsine-transformed) percentage of choice (approximate subtraction)** | | | | | |
| Response category | 19.18 | 4, 628 | < .001 | .89 | .07 |
| Range | < 1 | 1, 157 | > .1 | - | < .01 |
| Age | < 1 | 4, 157 | > .1 | - | < .01 |
| Response category X Range | 2.07 | 4, 628 | .087 | .95 | < .01 |
| Response category X Age | 2.02 | 16, 628 | .014 | .89 | .03 |
| Range X Age | 1.29 | 4, 157 | > .1 | - | < .01 |
| Response category X Range X Age | < 1 | 16, 628 | > .1 | .95 | < .01 |
| **Mean chosen response (approximate calculation)** | | | | | |
| Correct outcome | 1685.80 | 3, 471 | < .001 | .60 | .76 |
| Operation | 93.49 | 1, 157 | < .001 | - | .12 |
| Age | < 1 | 4, 157 | > .1 | - | < .01 |
| Correct outcome X Operation | 131.81 | 3, 471 | < .001 | .72 | .12 |
| Correct outcome X Age | 2.03 | 12, 471 | .049 | .60 | .01 |
| Operation X Age | 6.24 | 4, 157 | < .001 | - | .04 |
| Correct outcome X Operation X Age | 2.78 | 12, 471 | .004 | .72 | .01 |
| **Standard deviation (approximate calculation)** | | | | | |
| Correct outcome | 257.66 | 3, 471 | < .001 | .82 | .35 |
| Operation | 2.41 | 1, 157 | > .1 | - | < .01 |
| Age | 1.12 | 4, 157 | > .1 | - | < .01 |
| Correct outcome X Operation | 18.17 | 3, 471 | < .001 | .88 | .02 |
| Correct outcome X Age | 1.20 | 12, 471 | > .1 | .82 | < .01 |
| Operation X Age | < 1 | 4, 157 | > .1 | - | < .01 |
| Correct outcome X Operation X Age | 1.62 | 12, 471 | .095 | .88 | < .01 |
| **CV (approximate calculation)** | | | | | |
| Correct outcome | 5.88 | 3, 471 | < .001 | .90 | .01 |
| Operation | 30.28 | 1, 157 | < .001 | - | .03 |
| Age | 1.24 | 4, 157 | > .1 | - | < .01 |
| Correct outcome X Operation | 7.46 | 3, 471 | < .001 | .96 | .01 |
| Correct outcome X Age | 1.36 | 12, 471 | > .1 | .90 | .01 |
| Operation X Age | < 1 | 4, 157 | > .1 | - | < .01 |
| Correct outcome X Operation X Age | 1.56 | 12, 471 | > .1 | .96 | .01 |
| **Mean response bias (approximate calculation)** | | | | | |
| Operation | 60.20 | 1, 157 | < .001 | - | .17 |
| Age | < 1 | 4, 157 | > .1 | - | < .01 |
| Operation X Age | 4.45 | 4, 157 | .002 | - | .06 |
